# Optimisation of children z-score calculation based on new statistical techniques

**DOI:** 10.1371/journal.pone.0208362

**Published:** 2018-12-20

**Authors:** Antonio Martinez-Millana, Jessie M. Hulst, Mieke Boon, Peter Witters, Carlos Fernandez-Llatas, Ines Asseiceira, Joaquin Calvo-Lerma, Ignacio Basagoiti, Vicente Traver, Kris De Boeck, Carmen Ribes-Koninckx

**Affiliations:** 1 ITACA, Universitat Politècnica de València, Valencia, Spain; 2 Erasmus Medical Center, Sophia Children’s Hospital, Rotterdam, The Netherlands; 3 Department of Paediatrics, University Hospital of Leuven, University of Leuven, Leuven, Belgium; 4 Associação Portuguesa para a Investigação e Desenvolvimento da Faculdade de Medicina, Lisbon, Portugal; 5 Instituto de Investigación Sanitaria La Fe, Valencia, Spain; McMaster University, CANADA

## Abstract

**Background:**

Expressing anthropometric parameters (height, weight, BMI) as z-score is a key principle in the clinical assessment of children and adolescents. The Centre for Disease Control and Prevention (CDC) growth charts and the CDC-LMS method for z-score calculation are widely used to assess growth and nutritional status, though they can be imprecise in some percentiles.

**Objective:**

To improve the accuracy of z-score calculation by revising the statistical method using the original data used to develop current z-score calculators.

**Design:**

A Gaussian Process Regressions (GPR) was designed and internally validated. Z-scores for weight-for-age (WFA), height-for-age (HFA) and BMI-for-age (BMIFA) were compared with WHO and CDC-LMS methods in 1) standard z-score cut-off points, 2) simulated population of 3000 children and 3) real observations 212 children aged 2 to 18 yo.

**Results:**

GPR yielded more accurate calculation of z-scores for standard cut-off points (p<<0.001) with respect to CDC-LMS and WHO approaches. WFA, HFA and BMIFA z-score calculations based on the 3 different methods using simulated and real patients, showed a large variation irrespective of gender and age. Z-scores around 0 +/- 1 showed larger variation than the values above and below +/- 2.

**Conclusion:**

The revised z-score calculation method was more accurate than CDC-LMS and WHO methods for standard cut-off points. On simulated and real data, GPR based calculation provides more accurate z-score determinations, and thus, a better classification of patients below and above cut-off points. Statisticians and clinicians should consider the potential benefits of updating their calculation method for an accurate z-score determination.

## Introduction

The use of z-scores in medicine and paediatrics is widespread to accurately assess growth through anthropometric measurements such as height, weight and Body Mass Index (BMI). Using the most precise methods to calculate z-score is important because of the risk of misclassification and its additional consequences [1, 2].

Anthropometric measurements may have different distributions for different populations. Although curve-fitting may be imprecise, normal distributions are the most popular because they are scalable to the mean and standard deviation (SD). For a normal distribution, a z-score represents the distance in SDs of a given value to the mean value of the distribution. Z-score equal to 0 means an average value, while a z-score of +1 means the value is one SD above the mean value of the population. Z-score charts (also known as centile growth charts) are used in paediatric growth follow-up and to compare anthropometrical variables to detect the presence of malnutrition or disease [3].

WHO proposes the calculation of z-scores for the analysis and interpretation of anthropometric values either for population-based and individual assessment, and suggests z-scores as a sex-independent variable that can be grouped by combining sex and age groups. Moderate malnutrition is defined as a weight-for-age (WFA) between -3 and -2 SD below the mean of the WHO child growth standards. Similarly, moderate wasting (low weight-for-height (WFH)), stunting (low height-for-age (HFA)) are defined as z-score between -3 and -2 SD. Z-score values below -3 indicate severe wasting and stunting [4].

In 2002, the CDC published growth charts for several anthropometric measurements [5]. These charts are based on the National Health and Nutrition Examination Survey data from the 1960s through the 1980s to determine the distribution of height, weight and BMI in children, which varies by age and sex. For values in the obesity range, BMI z-scores have been found unsatisfactory because the statistical method used to construct the growth charts compresses the *z*-score scale [6,7]. As an alternative, but less widely used, linear regression models have been proposed to estimate z-scores [8] and to identify implausible z-score values [9].

Historically, a variety of parametric and nonparametric methods were employed to determine z-score values, but such models did not allow the calculation of percentiles or equivalent z-scores for other than the selected smoothed percentiles [10]. In 1990, Cole proposed the LMS equation [11], a method for z-score and percentile determination. LMS parameters are coefficients estimated from growth data, smoothed and then computed to map the values to percentiles (and z-scores). LMS deals with skewed distributions by adjusting parameters, but it can lead to poor fitting on small population samples [12]. Fundamentals of this method were employed by the CDC [13] on an inverse approach for determining LMS parameters and percentiles-smoothing. The CDC-LMS method does not guarantee a good fit to the empirical data [10] and moreover the tails of the distribution (values below 3% and over 97%) are not used [14]. Still, the CDC-LMS method is widely used by software tools and clinicians rely on it to follow-up patients’ growth and nutritional status.

The eruption of big data and machine learning in health has contributed to the development and implementation of new mathematical models to support clinical decisions [15]. Due to the Gaussian nature of the anthropometric measurements [16], we propose a new tool for calculating z-scores for HFA, WFA and BMIFA by using a Gaussian Process Regressions (GPR) model, without smoothing the curves to empirical data. In this paper, we explain the derivation of the proposed model and provide a comparison of the two current z-score determination models, the model proposed by WHO (using first order statistical moments) and the approximation proposed by CDC (inspired on Cole’s LMS method [11]) with the model based on GPR.

## Material and methods

The methodology for defining the experiments was based on the guidelines for reporting mathematical models in clinical medicine [15, 17]. The goal was to elaborate a mathematical model for z-score calculation based on standard z-scores and observations for HFA, WFA and BMIFA data from male and female children, aged 0 to 240 months. Due to the continuous characteristic of the z-score value, only regression models were considered to elaborate the mathematical models. A regression model in statistics refers to the mathematical operation of estimating the relationship among variables with respect to a numerical output. This relationship is based on the estimation of unknown coefficients (*β*), which, applied to the set of input predictors (*X*), will estimate the output variable (*Y*) in which *Y* = *f*(*β*_1_,*X*_1_,*β*_2_,*X*_2_…*β_n_*,*X_n_*).

This section is organized as follows: sub-section 2.1 and 2.2 describe the methods proposed by WHO and CDC for the z-score calculation; subsection 2.3 describes the fundamentals and validation of GPR; sub-section 2.4 depicts the comparison methodology of the new method and the two former methods in three stages and sub-section 2.5 describes the generation of simulated data for the second stage comparison.

### Method proposed by the World Health Organization

Z-score expresses the number of standard deviations below or above the reference mean or median value for an anthropometric variable. A fixed z-score interval implies a fixed height or weight difference for children of a given age. For population-based uses, a major advantage is that a group of z-scores can be subjected to summary statistics such as the mean and standard deviation. The formula for calculating the z-score according to the WHO is *z-score* = (*X*-*m*)/*SD*, in which *X* is the observed value (height, weight or BMI), *m* and *SD* are the mean and standard deviation value of the distribution corresponding the reference population.

WHO Growth Standards and the 2007 WHO Growth references introduced a revision of the LMS method proposed by Cole [12] to accommodate the distributions of different anthropometric measurements for children below 60 months and estimate the z-score for WFA, HFA and BMIFA measurements [18]. In this study we used the method based on first order statistical moments.

### Method proposed by the Centre for Disease Control and Prevention

The CDC method to determine LMS coefficients [5] is different from the one proposed by Cole [11]. Cole’s LMS recommended a penalized likelihood estimation procedure to the raw data, whereas CDC-LMS approach smoothed curves for percentiles were generated first, to thereafter determine the parameters corresponding to the smoothed percentile curves.

In the first stage, CDC-LMS consisted of smoothed empirical percentiles with parametric and nonparametric regression procedures. In the second stage smoothed curves were transformed by the imputation of the median (M), the generalized coefficient of variation (S), and the power in the Box-Cox transformation (L). Continuous variables as the age, height and weight were discretised and labelled by periods of 1 month excluding birth. Whereas WHO method does not perform any data distribution tune and assumes the normal fit of anthropometrical measures, CDC-LMS method has a two-fold procedure for curve-fitting, data smoothing and imputation of parameters. The calculation formula is described in details elsewhere [5, 10].

### Gaussian Process Regressions

GPR are statistical models in which the observations occur in a continuous domain and can be defined by their second-order statistics [19]. GPR are flexible methods to model nonlinear regression problems because rather than providing a single regression function, they provide a posterior density over target data [20]. The main principle is that the observed data, t (z_1_,z_2_,…z_n_) has a Gaussian joint distribution which can be calculated by weighting the input parameters, which also have a Gaussian distribution. Instead of using intercepts and coefficients (as linear and non-linear regressions do), GPR defines a covariance matrix parameterized by hyper-parameters. In this way GPR makes a prediction based on the posterior distribution [21], expressing similarities between the predictors and the response. This approach is different from WHO and CDC-LMS method, as it does not smooth data nor transforms and dynamically adapts the first order statistical moments of the distributions in the dataset.

GPR models were trained and validated using the selected z-score tables proposed by the CDC [https://www.cdc.gov/growthcharts/zscore.htm], which contains 8694 data points corresponding to specific z-scores (-2, -1.5,-1,-0.5, 0, 0.5, 1, 1.5, 2) for WFA and HFA and 7812 for BMIFA from 3686 subjects. Among the most popular we considered the Squared Exponential Kernel, the Exponential Kernel, the Matern 5/2 and the Rational Quadratic Kernel [22].

Predictors (input variables) were gender (male/female), the age in months (from 0 to 240) and the observed measurement (kg, cm or kg/m^2^). Performance metrics were assessed by means of the coefficient determination (R2), the rooted median squared error (RMSE), the Mean Squared Error (MSE) and the Mean Absolute Error (MAE). GPR model based on a Squared Exponential kernel achieved the best classification with an R^2^ = 100% and RMSE = 0.01 and was used for comparison with WHO and CDC-LMS method (Table A in S1 File).

The mathematical modelling and validation was performed using the Regression Learner toolkit of Matlab 2017R using the Academic License in Universitat Politècnica de València.

### Performance comparison

The GPR z-score calculation model was compared to the method proposed by WHO and the method proposed by CDC in three stages: 1) Comparison of the determination of the three models and the standard z-scores determined by CDC tables. 2) Comparison of the GPR model with the CDC-LMS model on a simulated population of 3000 children. 3) Comparison of the GPR model with the CDC-LMS model on a dataset of 731 observations sampled in a population of 212 children, participating in the MyCyFAPP trial (95% statistical power; for a bilateral test to determine a minimum difference of 10% between the two models). Statistically significant difference in z-core calculations was tested with one-way analysis of variance at 95% of confidence interval. Non-parametric distributions were assessed using the Wilcoxon signed-rank test. Patients’ parents or guardians provided informed consent for participation. Absolute mean error was measured for the z-score determinations.

### Simulation of plausible and implausible observations

**Fig 1:**
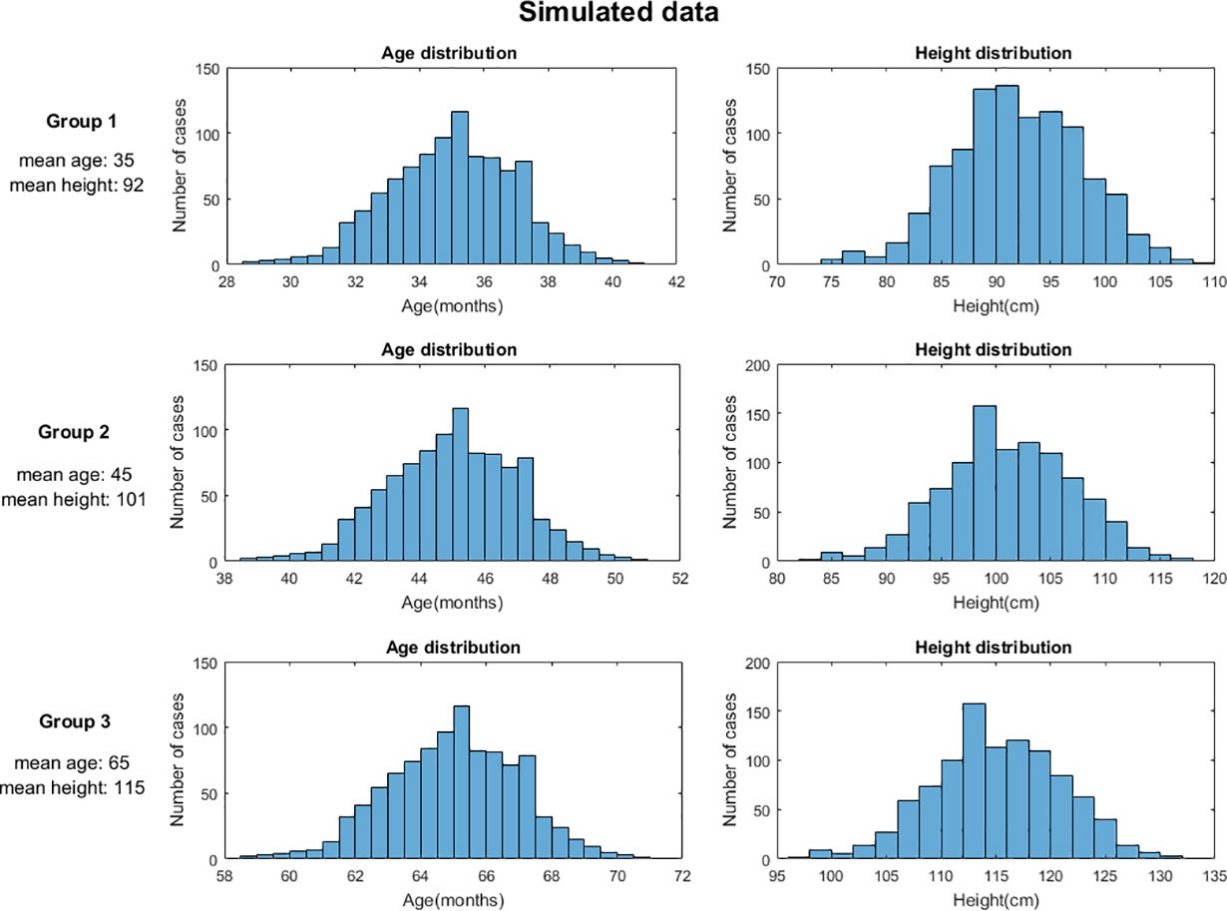
Simulated population for testing. Distribution of age and height observations for the randomly simulated cohort of three sub-population groups.

Plausible and implausible WFA, HFA and BMIFA observations were randomly generated using the Data Random Generation tool in Matlab2017. Three sub-population groups were generated at 3 randomly selected points: age 35, 45 and 65 months and a standard deviation of 10%; and height between 86 and 121 cm (Fig 1). A total of 3000 observations fitting a normal distribution was generated by providing first order statistical moments (mean and standard deviation). We chose these three sub-populations to test the performance of the z-score calculation methods when age-to-observation characteristics are modified.

## Results

### First stage comparison: Determination on standard points

Comparison among the z-scores determination using the WHO, CDC-LMS and GPR Squared Exponential model revealed a significant difference between the derived z-score values depending on the used method (p<0.001). Results for the comparison are discussed below, grouped by the anthropometric measurement (WFA in the main body of the manuscript; HFA and BMIFA for age in Figures A-D in S1 File).

**Fig 2:**
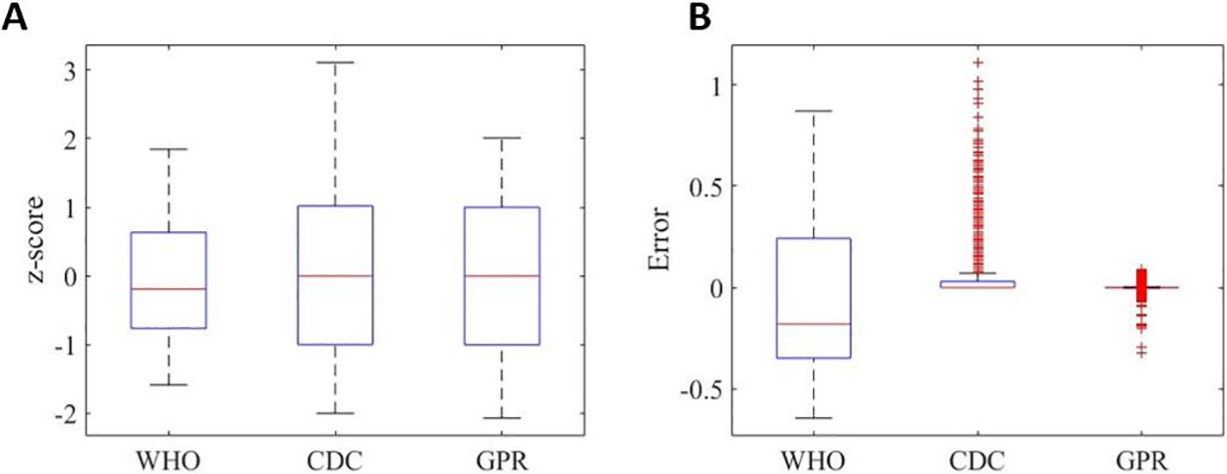
WFA z-score comparison for first stage. (A) WFA Z-score determination and (B) residuals comparison for the three models on 8694 data points from the CDC data set.

Z-scores for WFA values based on the standard data set from CDC were calculated using the 3 methods proposed by WHO, CDC-LMS and the GPR Squared Exponential and results are displayed in Fig 2. Fig 2A shows the box-whisker plot for the all-gender and all-ages output of the algorithm. Calculated values are in the expected range [-2, +2], except for CDC-LMS in which the upper whisker reaches +3 SD. Fig 2B shows the distribution of the residuals (actual z-score minus calculated z-score) for the three methods. The WHO method achieved residuals in the range of [-0.65, +0.90], whereas the CDC-LMS and GPR yielded better performance (p<<0.001). Residual outliers for the CDC-LMS method corresponded to children younger than 60 months.

**Fig 3:**
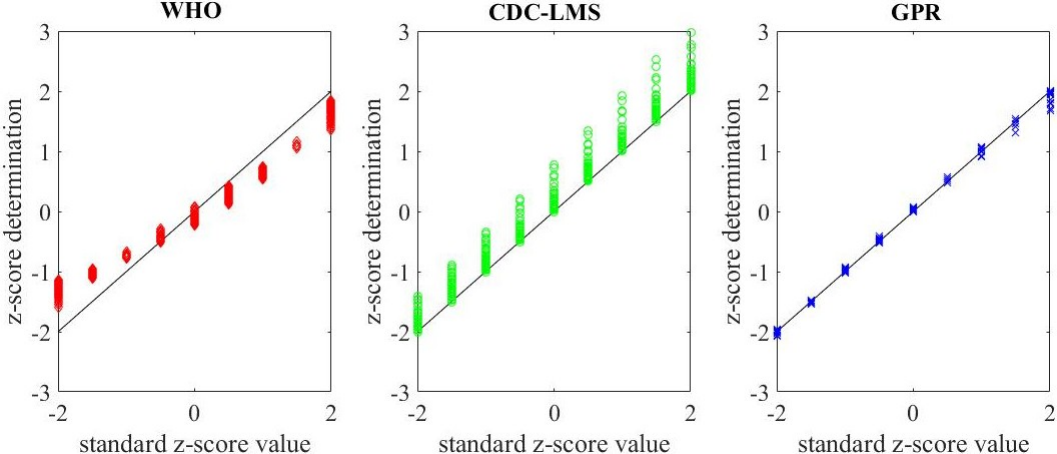
WFA z-score residuals for first stage. Z-score comparison for the three methods on 8694 WFA data points in CDC tables. Horizontal axis is for CDC tables’ z-score value. Calculated values (z-score determination in the vertical axis) are in red for WHO method, green for CDC-LMS method and blue for GPR method. Black slope for perfect fit.

**Fig 4:**
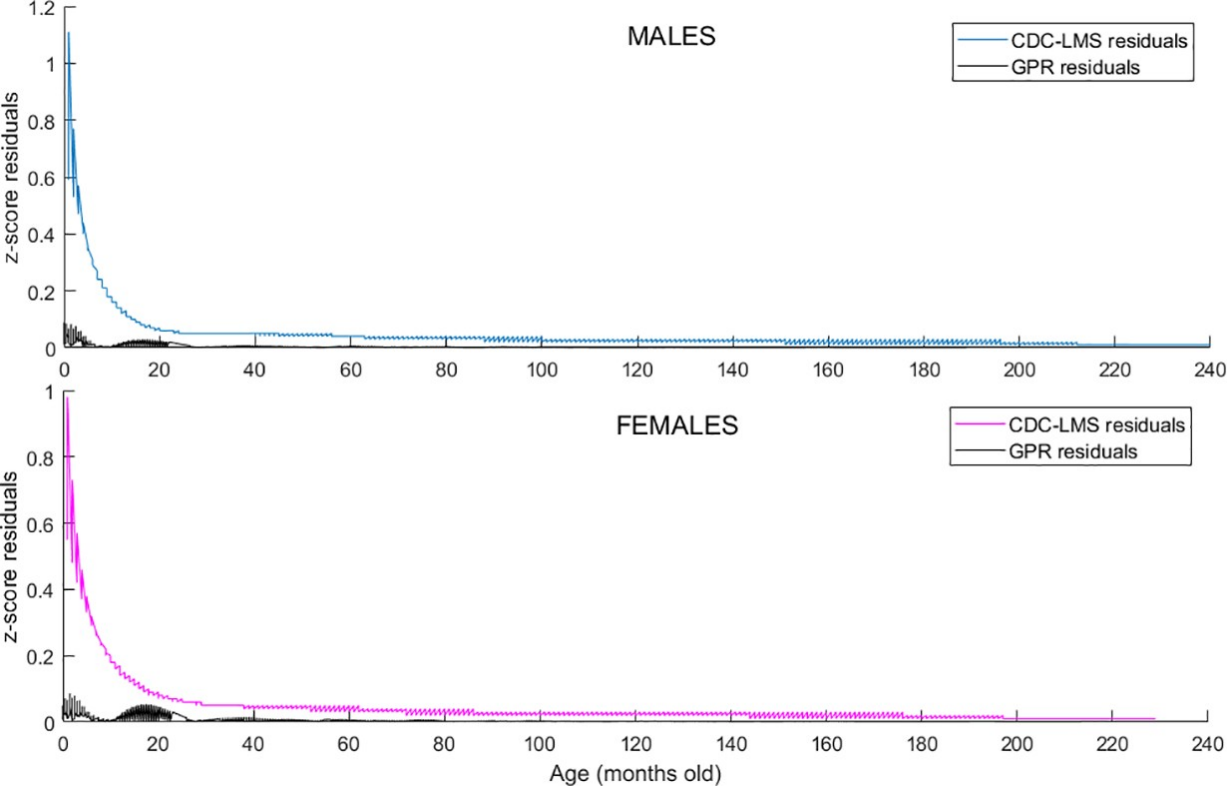
Gender-age influence in z-score estimation. Z-score residuals comparison for CDC-LMS and GRP methods for the 8694 WFA data points in CDC tables. Horizontal axis represents age of subjects in months. Residuals, calculated as standard z-score minus the calculated z-score for each method, is displayed in the vertical axis.

Fig 3 shows the comparison of the standard z-score value (horizontal axis) and the calculated value (vertical axis). The black slope represents the best possible calculation, in which the output of the model is the actual z-score value for the age and gender specific weight. The GPR model yields a better performance compared to the CDC-LMS and the WHO method for all z-score ranges. Fig 4 shows the behaviour of the calculation accuracy (residuals) per gender and age of subjects.

### Second stage comparison: Determination on simulated data

The second stage aimed to compare the calculation of z-scores in a simulated population of 3000 children (Fig 1). To this end, we compared the output of the CDC-LMS model and the GPR model for HFA values in a specific range of heights and ages (specifically below 60 months), in which the calculation of z-scores diverged for the two methods (Fig 4).

**Fig 5:**
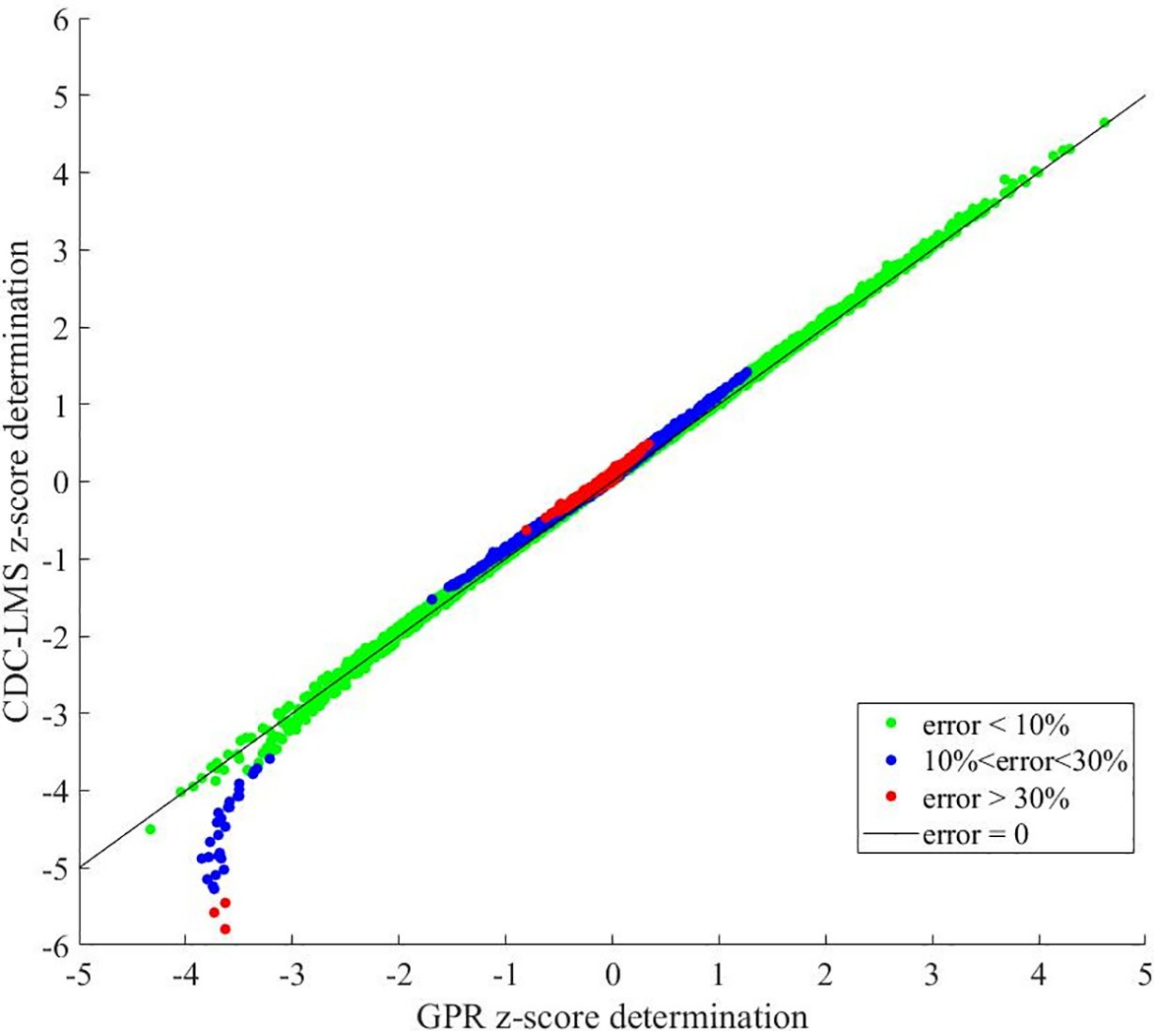
Comparative scatter plot of the calculated z-score for CDC-LMS method and the GPR method on the simulated population.

Error between calculated values using the two models was calculated as the arithmetic distance (difference among the two values) divided by the CDC-LMS value. Fig 5 shows the scatter plot of the absolute error for all the measurements. Divergence in the imputation of z-score was heterogeneous, for instance CDC-LMS calculated a z-score under -5 whereas GPR calculated z-scores higher than -4 for the same simulated patients. Overall, 11.1% of the comparisons had more than 30% of Absolute Error, and the majority of them were located in the z-score interval [–1, 1]. Errors lower than 30% but higher than 10%, were found in 24.5% of the comparisons and were located in the [–2, 2] interval.

### Third stage comparison: Determination on sampled data

The third and final stage consisted of comparing CDC-LMS and GPR models on 731 explorations (WFA and HFA) for 212 children who were regularly followed in the cystic fibrosis centres participating in the MyCyFAPP study (WP6.1 observational study)[23]. WHO method was not included in this study due to the underperformance shown in the 2 previous stages. Table 1 shows the descriptive statistics of this population for Gender, Weight, Height, Age and BMI for the third stage comparison.

**Table 1:** Descriptive values of stage 3 evaluation.

**Gender**	N, %	112, 52.8%	100, 47.2%
**Weight (Kg)**	Mean (SD)^a^	33.2 (16.2)	31.5 (14.4)
**Height (cm)**	Mean (SD)	135.2 (28.1)	132.46 (22.9)
**Age (month)**	Mean (SD)	117.65 (59.2)	115.24 (51.3)
**BMI**	Mean (SD)	16.91 (2.1)	16.84 (2.6)

^a^ Continuous variables are shown as mean (standard deviation).

The mean and standard deviation of the z-scores calculated with the three models are described in Table 2. Although the average value for the group shows similarities, the dependency test confirms the different distribution of the calculated z-scores.

**Table 2:** Mean and standard deviation of the z-score values calculates with CDC-LMS and GPR methods.

z-scores		CDC-LMS	GPR	*p* ^a^
**WFA**	Mean (SD)	-0.3 (0.9)	-0.3 (0.9)	<0.001
**HFA**	Mean (SD)	-0.1 (1.1)	-0.2 (1.1)	0.007
**BMIFA**	Mean (SD)	-0.3 (0.9)	-0.3 (0.9)	<0.001

^a^ Independence was tested using a Wilcoxon signed-rank test.

**Fig 6:**
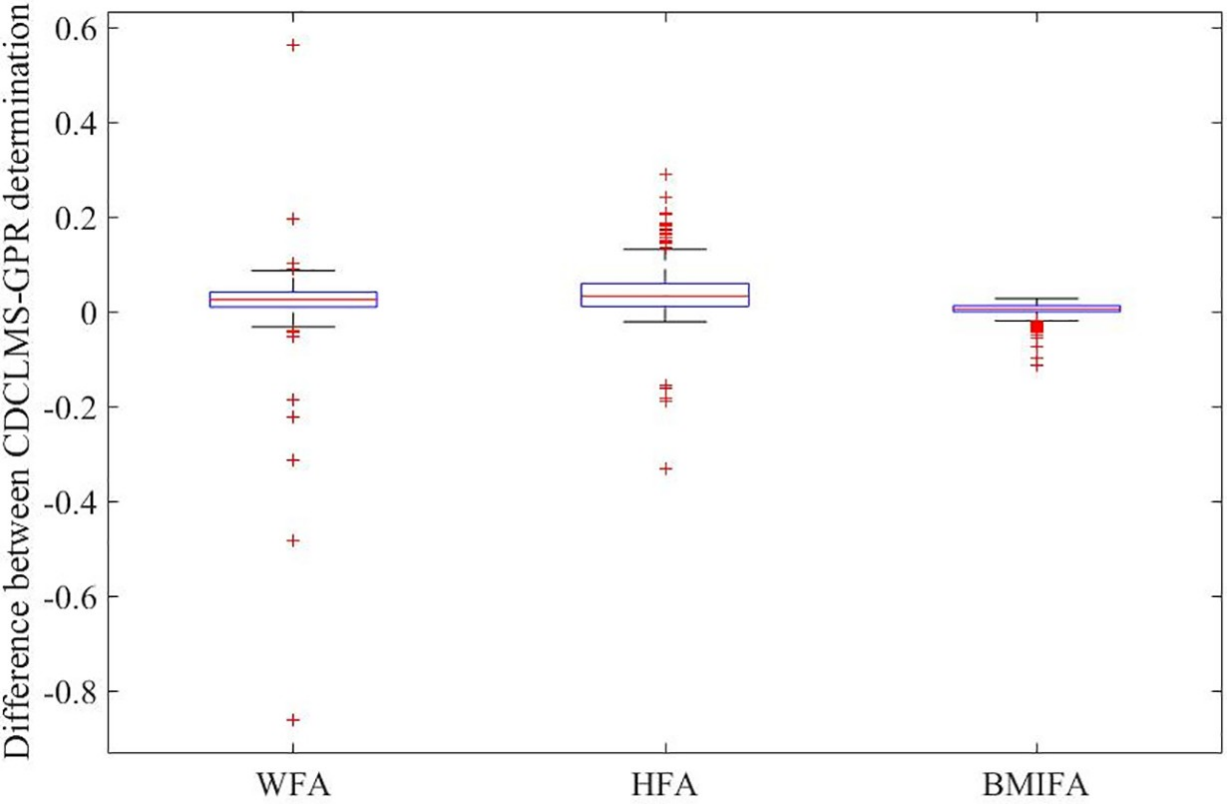
Z-score comparison for third stage. Differences between z-score determination based on CDC-LMS and GPR methods with real data (n = 731 physical explorations).

Fig 6 shows the distribution of the differences for WFA, HFA, and BMIFA z-scores based on the CDC-LMS method and the GPR method.

The mean differences are distributed near to zero, but differences in WFA z-scores have a range of 1.42 SD (min -0.86; max 0.56), with a narrow interquartile range ([0.01–0.04]). Differences in HFA z-scores have a range of 0.62 SD (min –0.33; max 0.28), and the majority of the differences are in the Confidence Interval at 95% = [0.01–0.06]. Differences in BMIFA z-scores have a range of 0.14 SD (min –0.11; max 0.02), and the majority of the differences are in the Confidence Interval at 95% = [0–0.01]. Finally, one way analysis of variance test for WFA, HFA and BMIFA z-score calculation differences confirmed the independency of the variability (p<0.001).

**Fig 7:**
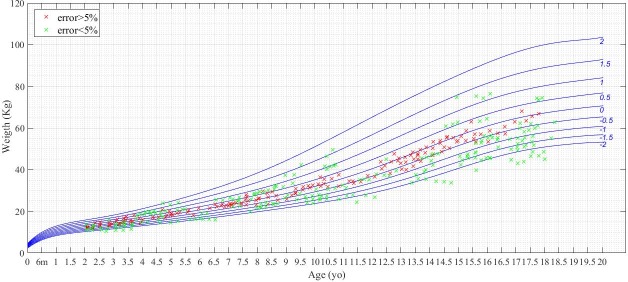
Weight determination for males of z-score in stage 3. Comparison of WFA z-score determination between CDC-LMS and GPR methods for males (n = 112). Green crosses are for determinations with less than 5% of error with respect to the CDC tables value. Red crosses are for determinations with an error above 5%. In blue, CDC standard growth curves.

**Fig 8:**
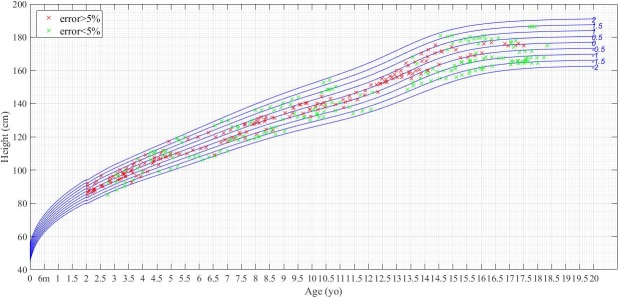
Height determination for males of z-score in stage 3. Comparison of HFA z-score determination between CDC-LMS and GPR methods for males (n = 112). Green crosses are for determinations with less than 5% of error with respect to the CDC tables’ value. Red crosses are for errors above 5%. In blue, CDC standard growth curves.

Figs 7 and 8 show the WFA and HFA data points allocated over the CDC standard growth charts respectively for males (charts for female may be found in Figures E and F in S1 File). These figures show in red the WFA and HFA observations in which the z-score calculation yielded an error greater than 5% between CDC-LMS and GPR method, and in green those with an error below or equal to 5%. To identify the distribution of the observations which are prone to have an error on its z-score determination, both figures include in blue the CDC standard growth curves. As observed in the simulated cohort, errors in the determination are more frequent in the range around z-score = 0 (in the central value of the distribution), whereas the values above and below 1SD yield less error. Even though the two calculation methods had similar mean and standard deviation, e.g.: -0.1 (1.1) for CDC-LMS versus -0.2 (1.1) for GPR in HFA, the individual determination of z-scores yielded different performance, p = 0.007 in HFA. Figures E and F in S1 File contain the same charts for females.

## Discussion

GPR yielded better performance than WHO and CDC-LMS methods for the calculation of z-scores in children for WFA, HFA and BMIFA (p<<0.001). To our knowledge, there are no previous studies comparing different methods for z-score calculation, being CDC-LMS a *de facto* standard. We demonstrated the use of a novel statistical approach to calculate z-scores for WFA, HFA and BMIFA in children under 20 years of age in three sets of data: standard CDC tables, simulated cohort of 3000 patients and a dataset of 731 observation from 212 children with CF (95% statistical power). Results confirm the accuracy of GPR on CDC data and a significant difference in the z-score calculation on simulated and real observations. Differences in z-score determination were independent of weight and BMI (p<0.01) and height (p = 0.007). Z-scores around the central value (z-score = 0) plus/minus one standard deviation (+/- 1SD) showed a significant difference in the z-score determination for WFA, HFA and BMIFA, hence concerning the majority of the population.

In assessing the methods to determine z-scores, we first looked into the LMS method introduced in the 1990 by Cole et al [11], and then looked at its adaptation to the CDC Growth Charts, which have modified this determination method and proposed new coefficients for z-score calculation [5]. In general, the changes concerned a transformation of the data for correcting the skewness of distribution at each age month, and then converting the percentile into z-score. The advance of new mathematical models and statistical tool-boxes in research allows researchers to propose new approaches to revise and optimize techniques and data. Various statistical methods and techniques have been used for curve-fitting and smoothing to help derive the related cut-off points for anthropometric measures in existing growth references and standards, and these methods/techniques can affect the derived cut points [10]. This paper presented the determination and performance comparison of a new method based on Gaussian Process Regression for z-score calculation. The main benefit of this new approach is that it does not transform data and adapts to the Gaussian distribution of the values, allowing a better performance of the z-score determination in standard cut-off points (Figs 2 and 3 and Fig 5; Table B and Figure G in S1 File). Our approach to deal with skewed distributions is to adjust the probability density function by means of first order statistical moments (weighted input parameters) instead of performing data smoothing as proposed by Cole in the LMS method [12].

Although we demonstrated that the GPR method is a practical approach to determine z-score in WFA, HFA and BMIFA, a Gaussian distribution was assumed, which may not reflect the shape of the growth trajectory and limit the results of this paper. This is the case since curve fitting is always based on a model and therefore may be imprecise and lead to misclassification. Moreover, our results should be confirmed in large longitudinal databases, like the National Health and Nutritional Examination Survey (NHANES https://www.cdc.gov/nchs/nhanes/index.htm)

Large organizations like WHO and CDC keep using the LMS method because it has been shown to be the best way to precisely calculate z-scores and its general acceptability in the clinical community [24]. Correct estimation of anthropometric z-scores is relevant for clinical patients´ care as well as for clinical studies since these z-scores are used for the classification of undernutrition and obesity with implications for treatment and/or nutritional intervention. They are also often used as outcome parameter in clinical studies and usually are included as part of more complex scoring systems for evaluating presence or progression of disease. For instance, the Nijmegen CDG paediatric rating scale [25], uses a z-score for scoring the Congenital disorders of glycosylation severity. Also, BMI z-score is used, together with serum biomarkers, in formulas to detect liver fibrosis and steatosis in children with chronic hepatitis C [26]. Moreover, z-scores are often used for the calculation of predictors for disease-specific risk scores and prognostic markers in paediatric disorders and in these circumstances [26], even a small difference in the z-score determination can lead the risk-score to underperform in the risk estimation. In addition, z-scores and percentiles have been used to classify other health related indicators, such as an increased blood pressure [27]. To further validate the anticipated improvement by the GPR method, the degree of tracking for z-scores for WFA, HFA and BMIFA in country specific longitudinal data sets could be of use for an accurate and precise z-score calculation.

## Conclusion

The use of GPR provides an accurate and precise model to determine z-scores for anthropometric measures in the paediatric population. The correct calculation of z-scores can increase the precision of evaluating growth and nutritional status and of calculating risk scores that are based on z-score values.

## Supporting information

S1 File Supporting information. Supporting information containing individually cited Tables and Figures.
(DOCX)
