# Supplementary material for: Optimisation of children z-score calculation based on new statistical techniques
Source: PLoS One. 2018 Dec 20;13(12):e0208362. doi: 10.1371/journal.pone.0208362 (PMC6301782; doi:10.1371/journal.pone.0208362)
Supplement: S1 File — Supporting information containing individually cited Tables and Figures. (DOCX) [file pone.0208362.s001.docx]

# Supporting information

**Table A.** **Performance of the Gaussian Process Regression kernel functions.**

| Observation | Kernel function | R^2 a^ | RMSE^b^ | MSE^c^ | MAE^d^ |
| --- | --- | --- | --- | --- | --- |
| BMI | Rational Quadratic | 1.00 | 0.00 | 0.00 | 0.00 |
|  | **Squared Exponential** | **1.00** | **0.00** | **0.00** | **0.00** |
|  | Matern 5/2 | 1.00 | 0.00 | 0.00 | 0.00 |
|  | Exponential | 1.00 | 0.01 | 0.00 | 0.01 |
| Height | Rational Quadratic | 1.00 | 0.02 | 0.00 | 0.01 |
|  | **Squared Exponential** | **1.00** | **0.01** | **0.00** | **0.01** |
|  | Matern 5/2 | 1.00 | 0.03 | 0.00 | 0.01 |
|  | Exponential | 1.00 | 0.04 | 0.00 | 0.01 |
| Weight | Rational Quadratic | 1.00 | 0.01 | 0.00 | 0.00 |
|  | **Squared Exponential** | **1.00** | **0.01** | **0.00** | **0.00** |
|  | Exponential | 1.00 | 0.02 | 0.00 | 0.00 |

^a^ R^2^: R-Squared; ^b^ RMSE: Rooted Median Squared Error; ^c^MSE: Mean Squared Error; ^d^ MAE: Mean Absolute Error


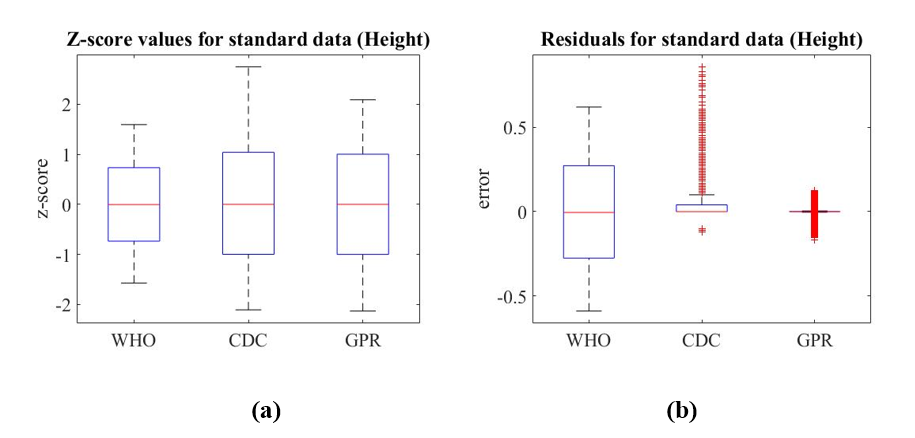


**Fig A**. **HFA z-score comparison for first stage**. Height z-score determination (a) and residuals (b) comparison for the three models.


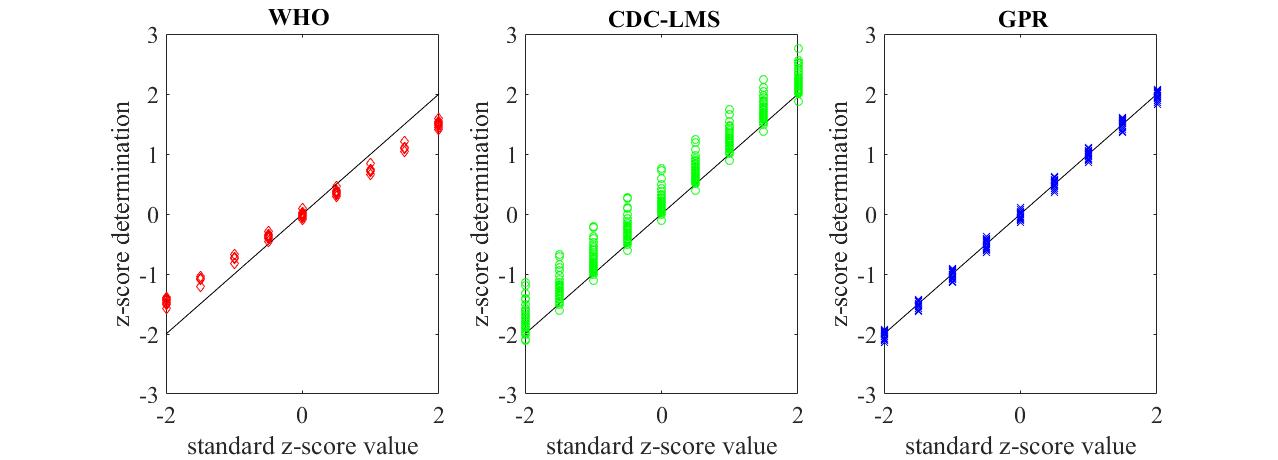


**Fig B HFA z-score residuals for first stage.** Z-score comparison using the three methods and standard z-score for 8694 HFA data points in CDC tables. Horizontal axis represents the standard z-score value from CDC tables. Calculated values (z-score determination in the vertical axis) are in red for WHO method, green for CDC-LMS method and blue for GPR method. Black slope is the perfect fit between expected value and calculated value.


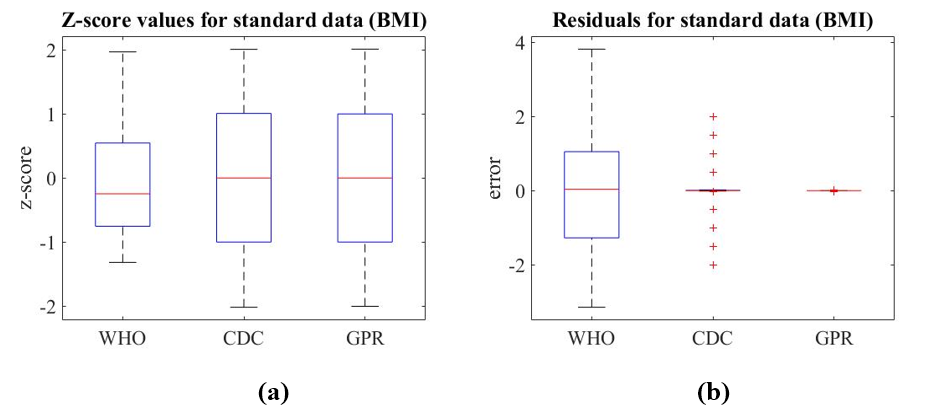


**Fig C. BMIFA z-score comparison for first stage.** BMIFA z-score determination (a) and residuals (b) comparison for the three models.


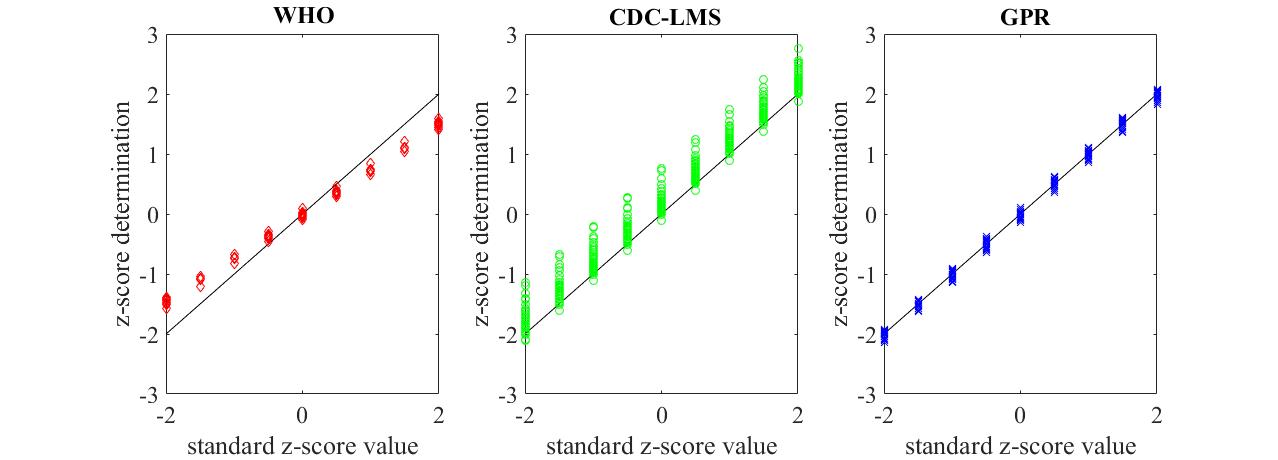


**Fig D.** **BMIFA z-score residuals for first stage**. Z-score comparison using the three methods and standard z-score for 7812 BMIFA data points in CDC tables. Horizontal axis represents the standard z-score value from CDC tables. Calculated values (z-score determination in the vertical axis) are in red for WHO method, green for CDC-LMS method and blue for GPR method. Black slope is the perfect fit between expected value and calculated value.


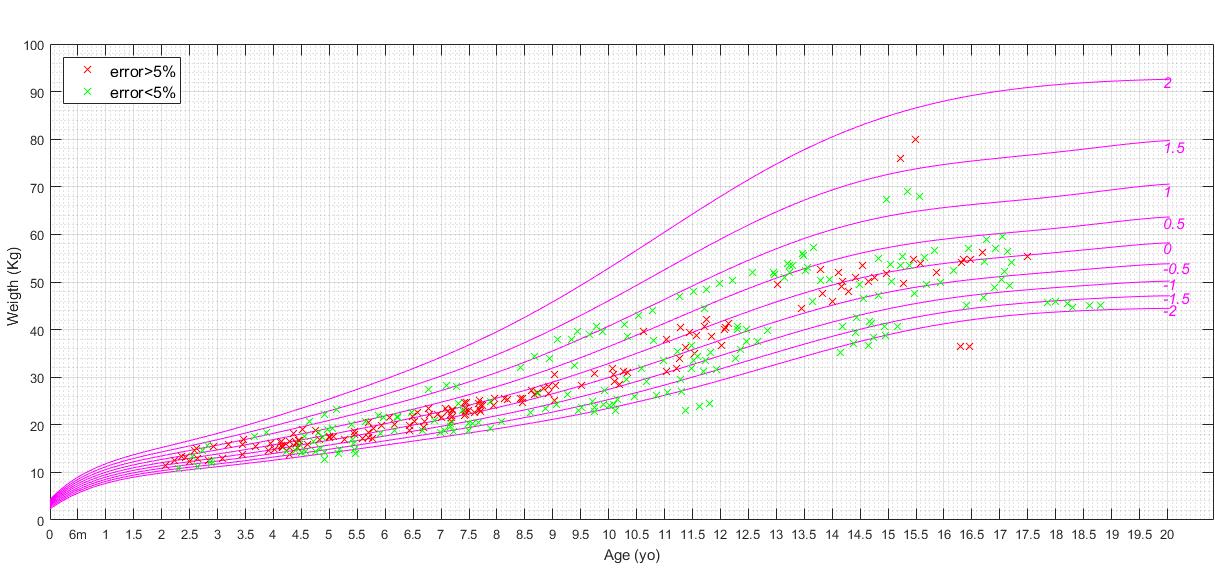


**Fig E.** **Weight determination for females of z-score in stage 3.** Comparison of WFA z-score determination between CDC-LMS and GPR methods for females. Green crosses are for determinations with less than 5% of error. Red crosses are for determinations with an error above 5%. CDC standard growth curves (pink) are included as a reference of the distribution of the observations with and without errors.

**
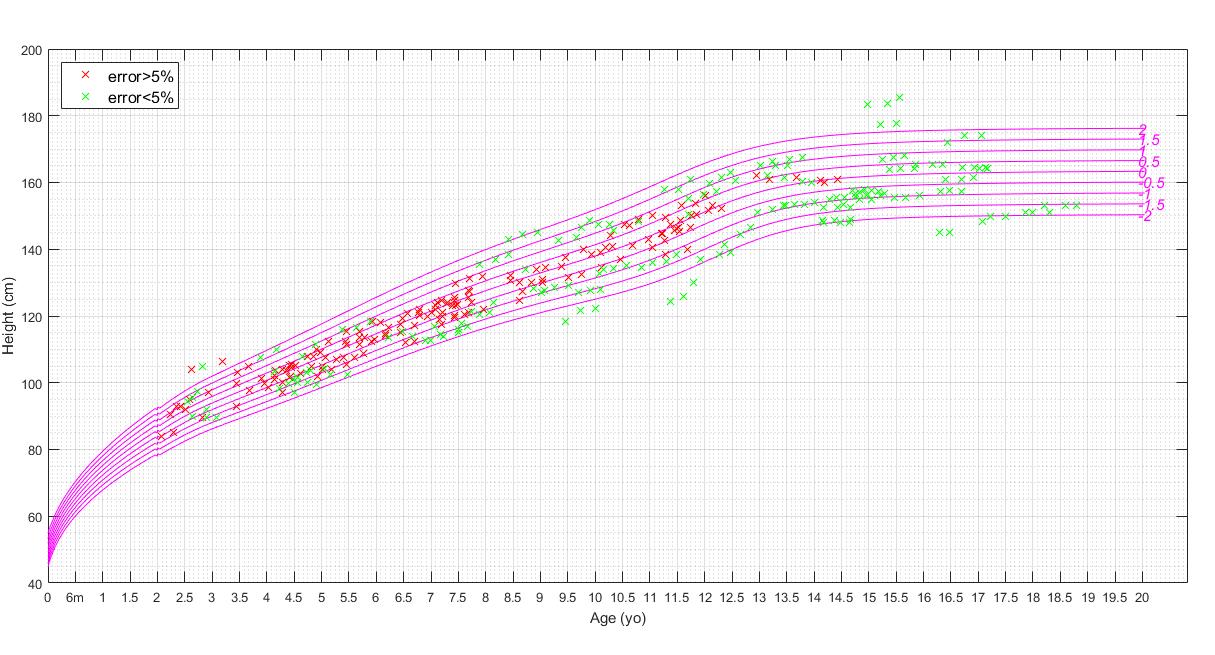
**

**Fig F. Height determination for females of z-score in stage 3.** Comparison of HFA z-score determination between CDC-LMS and GPR methods for females. Green crosses are for determinations with less than 5% of error. Red crosses are for determinations with an error above 5%. CDC standard growth curves (pinks) are included as a reference of the distribution of the observations with and without errors.

**Table B.** **Comparison of GPR and CDC-LMS models in the classification of nutritional ranges for malnourishment assessment.**

|  | | **CDC-LMS** | | |
| --- | --- | --- | --- | --- |
|  |  | [-1,-2] | [-2,-3] | [-3,-4] |
| **GPR** | [-1,-2] | 333 | 1 | 0 |
|  | [-2,-3] | 46 | 209 | 2 |
|  | [-3,-4] | 0 | 10 | 71 |
| Misclassification (%) | | **12.1** | **5.5** | **2.7** |


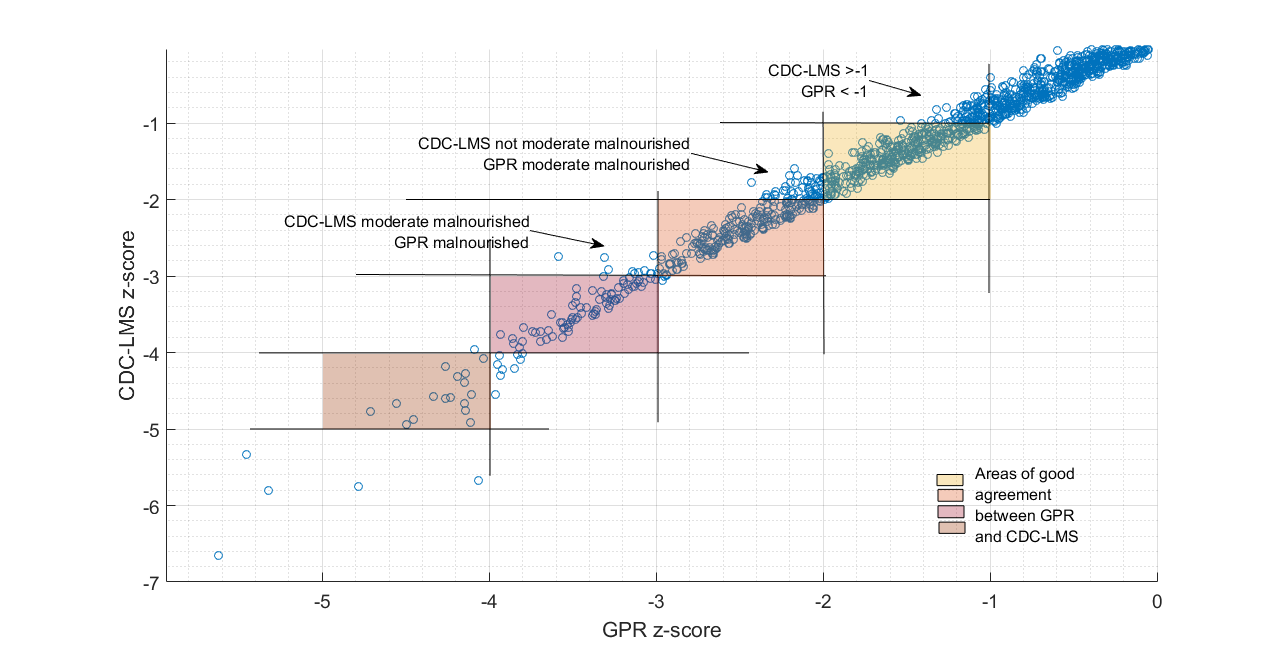


**Fig G. Determination of nutritional status.** Graphical representation of the comparison between GPR and CDC-LMS z-score estimators. Areas of agreement between the two models are shaded, whereas, the points of disagreement are left in white background.
